# A multiplex culture system for the long‐term growth of fission yeast cells

**DOI:** 10.1002/yea.3237

**Published:** 2017-06-06

**Authors:** Céline Callens, Nelson C. Coelho, Aaron W. Miller, Maria Rosa Domingo Sananes, Maitreya J. Dunham, Matthieu Denoual, Damien Coudreuse

**Affiliations:** ^1^ SyntheCell Team, Institute of Genetics and Development of Rennes CNRS UMR 6290 Rennes France; ^2^ Department of Genome Sciences University of Washington Seattle Washington USA; ^3^ Ecole National Supérieure d'Ingénieurs de Caen UMR 6072 – GREYC Caen France

**Keywords:** *Schizosaccharomyces*, continuous culture, experimental evolution, chemostats, turbidostats

## Abstract

Maintenance of long‐term cultures of yeast cells is central to a broad range of investigations, from metabolic studies to laboratory evolution assays. However, repeated dilutions of batch cultures lead to variations in medium composition, with implications for cell physiology. In Saccharomyces cerevisiae, powerful miniaturized chemostat setups, or ministat arrays, have been shown to allow for constant dilution of multiple independent cultures. Here we set out to adapt these arrays for continuous culture of a morphologically and physiologically distinct yeast, the fission yeast *Schizosaccharomyces pombe*, with the goal of maintaining constant population density over time. First, we demonstrated that the original ministats are incompatible with growing fission yeast for more than a few generations, prompting us to modify different aspects of the system design. Next, we identified critical parameters for sustaining unbiased vegetative growth in these conditions. This requires deletion of the *gsf2* flocculin‐encoding gene, along with addition of galactose to the medium and lowering of the culture temperature. Importantly, we improved the flexibility of the ministats by developing a piezo‐pump module for the independent regulation of the dilution rate of each culture. This made it possible to easily grow strains that have different generation times in the same assay. Our system therefore allows for maintaining multiple fission yeast cultures in exponential growth, adapting the dilution of each culture over time to keep constant population density for hundreds of generations. These multiplex culture systems open the door to a new range of long‐term experiments using this model organism. © 2017 The Authors. Yeast published by John Wiley & Sons, Ltd.

## Introduction

Experimental approaches using yeast as a model system generally rely on following batch cultures of cells over a few hours. In these conditions, cells are usually maintained in exponential growth, with densities that span a narrow range of values, avoiding nutritional stress or over‐dilution. In this context, longer‐term experiments are difficult to perform, in particular when cells must be maintained in vegetative growth. They involve daily dilutions of the cultures, which result in significant oscillations in the densities of the populations during the course of an experiment. This has strong implications for cell physiology, as cells are experiencing constantly changing growth conditions, in particular nutritional status. This can lead to specific cellular responses such as signalling through various stress pathways (Broach, 2012; de Nadal *et al*., 2011). Importantly, such responses to repeated changes in the cellular environment are likely to introduce biases that can alter the outcome of long‐term assays.

To circumvent these issues, yeast biologists have used a range of continuous culture systems, including chemostats and turbidostats (Nasmyth, 1979; de Jong‐Gubbels *et al*., 1996; Hoskisson and Hobbs, 2005; Klein *et al*., 2013; Miller *et al*., 2013; Takahashi *et al*., 2015). Such devices allow for better control, in some cases automated (Takahashi *et al*., 2015), of the conditions in which the cells are grown. A number of commercial products are also available that offer not only tight regulation of the cellular environment but also a number of real‐time measurements such as density or pH. However, such setups are expensive, difficult to manipulate and largely incompatible with large‐scale multiplex experiments, in which several replicates must be conducted in parallel and/or where multiple strains and conditions need to be simultaneously tested. In the last few years, custom‐made systems have been designed that are adapted to large‐scale experiments in lower volume in a controlled manner. From the ‘ministat’ chemostat arrays (Miller *et al*., 2013) to more advanced turbidostat setups (Takahashi *et al*., 2015), a range of powerful devices have been described that are cost‐effective, do not present the drawbacks mentioned above and can generally be set up in any biology laboratory without highly specific expertise. In eukaryotes, such systems have mostly been used with the budding yeast Saccharomyces cerevisiae for a range of experiments (Payen *et al*., 2014; Sunshine *et al*., 2015; Keren *et al*., 2015). However, such devices have clear potential to be used in any type of assay that requires long‐term, stable growing conditions. These include, but are not restricted to, competition assays, long‐term responses of cells to different levels of stress, investigations of genome alterations over time in exponentially growing cells and aging assays. It is, however, unclear whether these setups, initially designed for small‐sized budding yeast cells, can be used as such with other, bigger yeasts with very different physiologies.

In this study, we have adapted the ministats to the use of the fission yeast *Schizosaccharomyces pombe*, with the goal of maintaining cultures in exponential growth at constant density over long periods of time through modulation of the dilution rates. We first show that the original ministat system is not compatible with fission yeast, as phenotypes such as flocculation or cell adherence were found to reduce the maximum duration of any experiment to only a few days. This prompted us to modify the setup and alter both the growth medium and genetic background of the cells, making it possible to perform experiments that can last several hundreds of generations. In addition, we developed a novel and simple feeding system based on micropiezo pumps, which allows for individual control of the rate at which each individual culture in an array of ministats is diluted. This is an appealing property of such a versatile device, as it facilitates the parallel growth of strains that can have very different generation times or the simultaneous use of different culture media in which cell growth is altered. The proposed setup is therefore compatible with multiplexed long‐term experiments using exponentially growing fission yeast cells.

## Materials and methods

### Fission yeast strains and methods

Standard media and methods were used (Hayles and Nurse, 1992; Moreno *et al*., 1991). Strains used in this study were PN1 (*972 h* ^−^), DC477(*gsf2*Δ*::KAN h* ^−^), DC561 (*leu1::Pcdc13::cdc13Scdc2as::cdc13UTR::ura4* ^*+*^ *cdc2*Δ*::KAN cdc13*Δ*::NAT cig1*Δ*::HYG cig2*Δ*::KAN puc1*Δ*::leu2* ^*+*^ *gsf2*Δ*::KAN ura4‐D18 h* ^*+*^; referred to as *MCN gsf2*Δ) and DC566 (*leu1::Pcdc13::cdc13Scdc2T14AY15Fas::cdc13UTR::ura4* ^*+*^ *cdc2*Δ*::KAN cdc13*Δ*::NAT cig1*Δ*::HYG cig2*Δ*::KAN puc1*Δ*::leu2* ^*+*^ *gsf2*Δ*::KAN ura4‐D18 h* ^−^; referred to as *MCNAF gsf2*Δ). *gsf2*Δ*::KAN* is a complete deletion of the open reading frame generated by homologous recombination of a kanamycin resistance cassette. Cells operating with the Cdc13‐Cdc2 fusions have been previously described (Coudreuse and Nurse, 2010). For S. japonicus, the wild‐type NIG2028 (*matsj‐P2028 h* ^*−*^) strain was used. Experiments were carried out in yeast extract complex medium supplemented with 0.225 mg/mL of l‐histidine, l‐leucine, adenine and uridine (YE4S) or in minimal medium supplemented with 0.225 mg/mL of l‐histidine, l‐leucine, adenine, uridine, l‐lysine and l‐arginine (EMM6S). Galactose (D+ galactose, Sigma Aldrich) was added where indicated at a final concentration of 200 mm (3%).

### Ministat setups using a multichannel peristaltic pump

**Figure 1:**
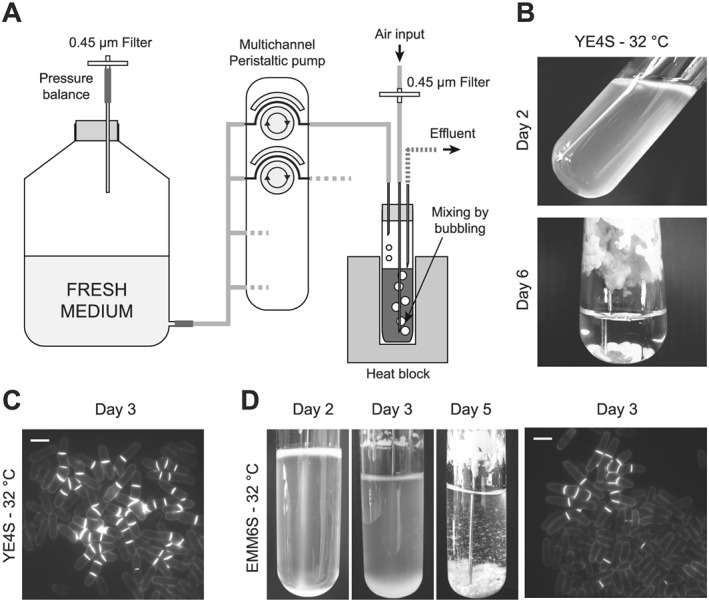
Flocculation and division phenotypes using *Schizosaccharomyces pombe* in ministat setups. (A) Schematic representation of the initial ministat system from Miller *et al*. (2013). (B) *S. pombe* cells were inoculated at an optical density of 0.4 (595 nm) in standard ministats in rich medium (YE4S) at 32°C. While the initial generations produced normal cells, we observed complete flocculation after 5–6 days. (C) Rapid appearance of multiseptated and branched cells that took over the entire population after 6 days. Blankophor staining of a representative sample at day 3 of the experiment. Scale bar = 10 μm. (D) Ministat experiments were conducted in minimal medium (EMM6S) at 32°C. Again, flocculation was observed (left panels, days 2, 3 and 5) and aberrant cells appeared in the cultures, although at a later time than in rich medium (right panel; blankophor staining of a representative sample at day 3 of the experiment). Scale bar = 10 μm. Note the changes in volume during the first few days of the experiment. These result from adjustment of the position of the effluent needle in order to stabilize the population density, which is particularly difficult to achieve when the cultures undergo strong flocculation.

**Figure 2:**
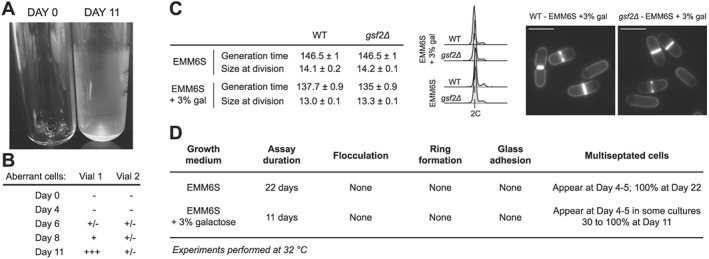
Addition of galactose and deletion of the flocculin‐encoding gene *gsf2*. (A) *S. pombe* cells were grown in the ministats as in Figure 1 in EMM6S supplemented with 3% galactose at 32°C. While this prevented flocculation, a ring of healthy cells accumulated at the top of the liquid column, introducing a strong bias for conducting any selection experiments in these devices (data not shown, similar to Figure 1D). In addition, we observed the formation of a thin film of cells adhering to the glass wall of the vessels, covering the entire surface in contact with the cell culture. A clean empty tube (day 0) is shown for comparison. The tube at day 11 was emptied, highlighting the presence of cells on the wall of the glass vessel. (B) The presence of galactose in these experiments did not prevent the random accumulation of aberrant cells in the device. The results for two independent cultures are shown. The scores were assigned as in Table 1. (C) Deletion of the *gsf2* flocculin‐encoding gene has no visible effect on cells exponentially growing in batch cultures in EMM6S or EMM6S + 3% galactose at 32°C. Left panel: generation time and size at division for the indicated strains and growth conditions. WT, Wild type. Standard errors of three independent experiments are shown. For cell size at division, *n* > 50 for each independent experiment. Middle panel: DNA content analysis. Right panel: blankophor staining for cultures in EMM6S + 3% galactose. Scale bar = 10 μm. (D) Characterization of ministat assays using *gsf*2Δ cells grown in the indicated media at 32°C.

**Figure 3:**
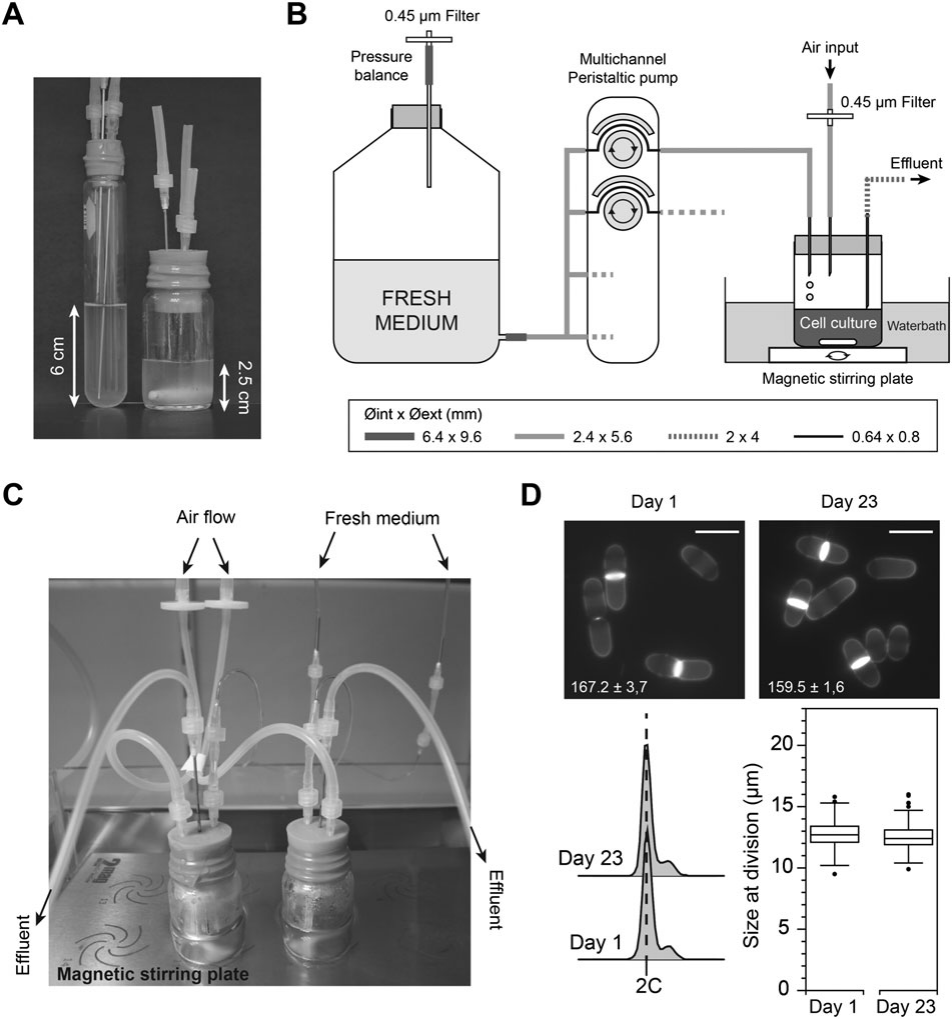
Increased mixing of the population prevents the accumulation of aberrant cells. (A) To limit the height of the liquid column in the system, thereby reducing size selection, we switched to using wider culture vessels. Left: initial ministat vial. Right: new ministat vial. Each vial contains 20 mL of cultures. The liquid column in the new vials is only 2.5 cm high, compared with 6 cm in the standard tubes. (B) Schematics of the entire system, in which the new vessels were maintained at the appropriate temperature by immersion in a temperature‐controlled water bath. Mixing of the cells was achieved using a waterproof magnetic stirring system. The diameters of the different tubings are indicated. (C) Image showing the assembled culture vials on the immersed multiposition stirring system. (D) *gsf*2Δ cells were maintained at a constant optical density of 0.4 (595 nm) in EMM6S + 3% galactose at 28°C for 23 days (>150 generations). To this end, the dilution rate was adjusted throughout the experiment, compensating for potential changes in growth rate (while the density is constant, improved growth would be reflected by increased dilution rate). At the end of the experiment, cells appeared healthy and we did not observe either flocculation or accumulation of multiseptated and branched cells. Top panels: blankophor staining of cells at day 1 and day 23. Scale bar = 10 μm. Generation times are indicated with standard errors of three independent cultures. Bottom panel, left: DNA content of cells at days 1 and 23. Bottom panel, right: box and whiskers plot of cell size at division (*n* > 100) at days 1 and 23.

The initial ministat setups (used for Figures 1 and 2) were as previously described (Miller *et al*., 2013). All tubings were silicon Tygon 3350 except those mounted in the peristaltic pump, which were marprene tubings (Watson Marlow 2015S/CA16). Needles used in the different setups were Air‐Tite hypodermic needles (16G × 5 inch; effluent line) and BD spinal needles (medium and air inputs; 18G × 6 inch). The manifolds for applying air pressure to multiple vessels from a unique air chamber were four‐port systems (180°, Cole Parmer). The peristaltic pump was a 32‐channel pump (205CA16 with a 16 channel extension 505CAX16, Watson Marlow). For adapting these systems to *S. pombe* cultures (Figure 3), 50 mL flat‐bottom glass bottles (Valumetric glass bottles, 2 oz, Wheaton) were used. To ensure efficient mixing of the cell cultures in these bottles, the module that initially allowed for both mixing and bubbling air through the cultures (Miller *et al*., 2013) was replaced by a multiposition magnetic stirring system (MIXdrive 15, 2mag AG) immersed in a temperature‐controlled water bath. Pressure was still maintained in the vials for the operation of the effluent lines using a standard aquarium pump (Rena Air 600), an air chamber and non‐immersed needles.

Yeast cells were grown exponentially overnight in batch cultures at 28 or 32°C according to the target temperature of the ministats. Aliquots of 20 mL of these cultures were then inoculated into each glass vessel at an initial optical density of 0.4–0.5 at 595 nm (~5–6 × 10^6^ cells/mL). Medium was then continuously provided to the cultures through the operation of the peristaltic pump. The starting rotation speed of the pump was determined by the initial average generation time of the different cultures and adjusted regularly over the course of the experiments in order to maintain them at constant density. When using strains that have different generation times, the volume of each culture was adjusted below or above 20 mL to adapt the dilution rate from a constant medium flow rate. This allowed us to maintained all the cultures at constant optical density despite different growth rates. For optical density measurements, samples were collected from the effluent lines and measured at 595 nm in an Amersham Ultraspec 2100 Pro spectrophotometer.

### Assays for preventing flocculation and glass adherence

For the various tests described in this study to solve the problems of flocculation and glass adherence, a panel of treatments was assessed by adding different chemicals to the medium at the concentrations indicated in Table 2: EDTA (Sigma Aldrich), phthalate (Sigma Aldrich) and a non‐ionic surfactant (10% Pluronic F‐68, Life Technologies). For the glass treatment with silane, the vials were washed with detergent, rinsed with water and dried. Each vial was then positioned upside‐down above narrow glass vials containing trichloromethyl‐silane (Acros Organics), and left overnight to allow full evaporation of the silane and its deposition on the glass walls. The tubes were then rinsed with hot water and detergent, rinsed again with water, dried overnight at 37°C and autoclaved.

### DNA content analysis

DNA content analyses were performed by flow cytometry. Cells were fixed in 70% ethanol, washed in 50 mm sodium citrate, treated with RNAse A (0.1 mg/mL) overnight at 37°C and stained with propidium iodide (2 μg/mL). DNA content was determined using a BD Accuri C6 flow cytometer and analysed using the FlowJo software. Note that fission yeast cells have a short G1 and DNA replication occurs prior to cytokinesis. Cells therefore have a 2C DNA content for most of their cell cycle. In asynchronous cultures, a small 4C peak can be observed since a small percentage of cells are binucleated and undergo DNA replication.

### Microscopy

All microscopy experiments were performed on an inverted Zeiss Axio Observer (Carl Zeiss Inc.) equipped with a Lumencor Spectra X illumination system and a Hamamatsu Orca Flash 4.0 V2 sCMOS camera. Images were acquired using Visiview (Visitron GmbH) and analysed using Fiji (National Institutes of Health). For the visualization of the cell wall and division septum, living cells were stained with Blankophor (MP Biochemicals). Size at division was determined from Blankophor stained cells using Fiji and the Pointpicker plug‐in.

### Ministat setups using individual piezo pumps

**Figure 4:**
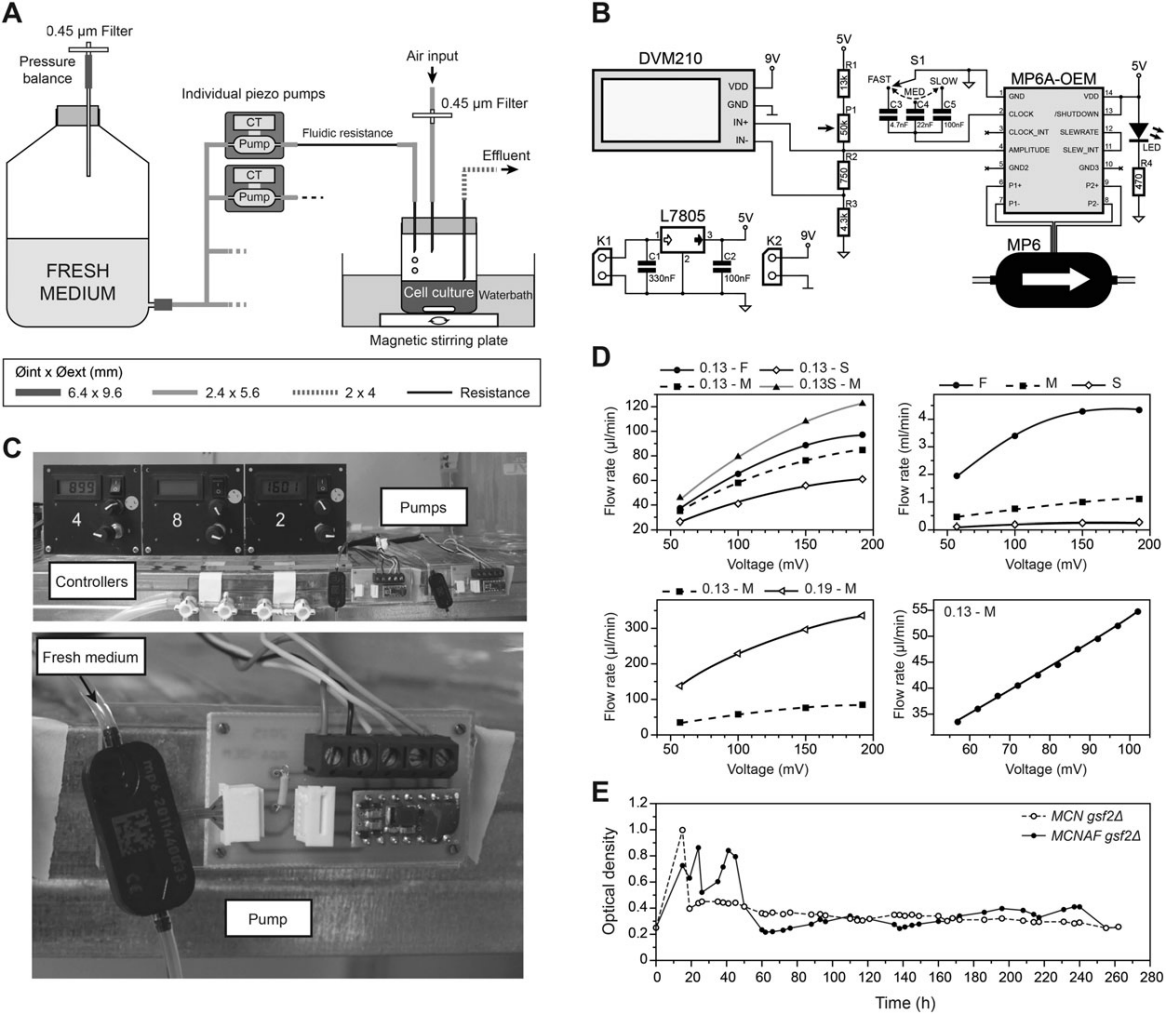
Development of a system for independent control of individual cultures. (A) Schematics of the new system integrating piezo pumps and their controllers (CT). A fluidic resistance downstream of each pump was required to reduce the flow rate to the appropriate range. The diameter of the different tubes is indicated. (B) Electronic scheme of the pump control system. MP6: piezo pump. See ‘Materials and methods’ for details of the system. (C) Representative images of the pump system. The top panel shows three control panels and two piezo pumps with their controllers. The bottom panel is a detailed view of a piezo pump and its electronic controller. (D) Changes in fluidic resistance allow for a broad range of flow rates. Top left: a 0.13 mm inner diameter resistance of 37 cm was used at fast (F), medium (M) and slow (S) frequencies for the pump. Shortening this resistance to 23 cm (0.13S–M) has a strong effect on flow rate. Top right: in the absence of fluidic resistance, very high flow rates can be obtained. Bottom left: changing the inner diameter of the resistance (0.13 vs. 0.19 mm here at medium frequency) has a strong effect on the flow rate. Bottom right: this system allows for fine modulation of the flow rate – shorter steps in voltage were used with a 0.13 mm fluidic resistance of 37 cm at a medium frequency, displaying a linear relationship between the imposed voltage and the flow rate. (E) Proof‐of‐concept experiment using the pump setup. 20 mL aliquots of *MCN gsf2*Δ and *MCNAF gsf2*Δ cells were grown in the new ministat system over 10 days (EMM6S + 3% galactose at 28°C), individually modulating the dilution rate of each culture throughout the experiment by only using the piezo pump parameters. After the usual first 48 h required for stabilization of the cultures, both strains were maintained at constant densities in exponential growth, despite their significantly different generation times (see main text).

To allow for a more normalized setup with standard and constant culture volumes, we developed a system that allows for precise individual control of the medium flow rate in each culture vessel. This system is based on commercially available piezo pumps that can be sterilized at high temperature (MP6 pumps, Bartels Mikrotechnik GmbH). To generate pumping modules specific to each culture, we fabricated individual electronic boards with a common power supply (Figure 4B).

The provided MP6‐OEM controller generates the high voltage (100–250 V) that is required for the actuation of the piezoelectric pump. The flow rate of the pump can be controlled through the amplitude and/or frequency of the input signal with linear dependencies for both parameters (Bartels Mikrotechnik GmbH, MP6 pump and controller manual). In this setup, the amplitude is tuned by a resistive potentiometer (P1) associated with resistors (R1, R2 and R3) in a voltage divider bridge. The values of the resistors were chosen to derive voltage amplitude at the input of the MP6‐OEM module in the range of 0.35–1.3 V, according to the manufacturer's instructions. The classically used single‐bottom resistor of the voltage divider bridge is here split into two resistors, R2 and R3, to allow differential measurement with a digital voltmeter module (DVM210) whose input range is 0–199.9 mV. The voltage displayed by the DVM210 is representative of/proportional to the amplitude of the input signal. A rotative knob (S1) allows for selection between three frequency ranges by switching between the three capacitors C3 (fast), C4 (medium) and C5 (slow). The MP6‐OEM module is powered at 5 V after a regulator (L7805 with associated capacitors C1 and C2). A 90‐264VAC‐9 V transformer (plugged to an AC line power) feeds the circuit board through the power supply connector K1. An LED is added with its current limiting resistor R4 as a power supply indicator. Each digital voltmeter module in the multiplex system is powered by a standard 9 V battery connected to power supply connector K2.

For adapting the range of flow rates to efficiently dilute the cultures, given the pumping rates of the piezo systems, fluidic resistances were added downstream of each pump using tubings of various lengths and internal diameters (Tygon tubing, Cole Parmer; see Figure 4D). The use of these pumps therefore allows for modulation of each dilution rate individually. The proof‐of‐concept experiments presented in Figure 4 were conducted using standardized setups, with constant volumes of cultures.

## Results and discussion

### Growth of *S. pombe* in ministat arrays

To automatically maintain multiple fission yeast cultures at constant density over long periods of time, we began by testing a previously described chemostat setup that was developed for the budding yeast (Miller *et al*., 2013; Figure 1A). These systems, referred to as ‘ministats’, rely on the use of a multichannel peristaltic pump that allows for slow renewal of the culture medium in each individual vial. For each sample, the volume of culture remains constant owing to the presence of an effluent line and the pressure inside the tube that is imposed by a permanent supply of air. Thus, the slow addition of fresh medium is compensated for by this effluent line, through which small volumes of the culture are discarded when the liquid column comes into contact with the effluent needle. The air input is also used to generate bubbling in the culture, thereby thoroughly aerating and mixing the population of cells. Finally, to ensure appropriate temperature of the samples, all of the vials are placed in highly controlled heat blocks. While these setups were previously used as chemostats, we surmised that they could also be adapted to maintain constant culture densities by regularly monitoring this parameter and adjusting dilution rates accordingly.

We initially grew 20 mL of wild‐type fission yeast cells in these ministats at 32°C in rich medium, with a target optical density at 595 nm of ~0.4 (~5 × 10^6^ cells/mL). While the first few generations appeared to be normal, we observed two distinct phenomena that were incompatible with our goal of maintaining cells in exponential growth over long periods of time: (a) after 5–6 days, all of the cultures had entirely flocculated, forming large aggregates of cells that accumulated at the bottom of the tubes and along the glass walls (Figure 1B); and (b) multiseptated and branched cells appeared after 3 days and took over the entire cultures after only 6 days (Figure 1C, Table 1). Interestingly, when performing the same assays in minimal medium (EMM6S), we observed differences in the behaviour of the cultures. Indeed, rings of cells rapidly accumulated at the top of the liquid columns (Figure 1D, day 3). As these cells are not equivalently diluted as those in the liquid culture, this potentially introduces a strong bias in the experiments. Furthermore, the appearance of aberrant cells was delayed (Table 1). However, flocculation was strong and occurred even earlier than with rich medium (Figure 1D).

**Table 1:** Accumulation of multiseptated and branched cells.

Time	YE4S	EMM6S
Day 0	−	−
Day 2	−	−
Day 3	+	±
Day 4	++	+
Day 5	n.d.	n.d.

Quantification of the branched and multiseptated cells in the experiments in Figure 1(B–D). These aberrant cells rapidly form clumps, making the precise determination of their percentage in the population difficult. We therefore assigned the following score: —, No aberrant cells; ±, 0–3%; +, 3–15%; ++, 15–45%; n.d., not determined. After 5 days, the flocculation of the cultures made it difficult to quantitatively assess the proportion of aberrant cells in the cultures. However, visual scoring suggested that multiseptated cells represented 100% of the population in YE4S at day 6, while normal cells were still present in EMM6S at that time point.

These initial assays suggested that (a) faster growth in rich medium may be incompatible with these long‐term cultures, (b) the accompanying phenotype may be amplified by the size selection occurring in these setups, giving advantage to the heavier, multiseptated and branched cells, (c) the use of the larger *S. pombe* cells compared with budding yeast may require a more efficient mixing to limit the size selection in the vials and (d) flocculation is a strong obstacle to performing long‐term experiments with *S. pombe* in the ministats.

### Addition of galactose efficiently prevents flocculation in the ministats

To adapt the ministats for the use of fission yeast, we first addressed the problem of flocculation in the cultures. Galactose has been shown to be a key player in the recognition mechanisms that bring about the surface interactions associated with the formation of flocs, and its addition to the medium inhibits flocculation (Tanaka *et al*., 1999; Matsuzawa *et al*., 2011, 2013). We used standard ministat setups to grow wild‐type cells at 32°C in minimal medium supplemented with 3% galactose. Interestingly, while this did not prevent the formation of a dense ring of cells at the upper limit of the liquid column within 5–7 days (data not shown), it strongly reduced flocculation over an 11 day experiment. This suggests that the addition of galactose to the medium may be sufficient to prevent the problem of flocculation for long‐term experiments. However, this identified another issue when using *S. pombe* in the ministat systems. Indeed, in addition to the ring mentioned above, we observed the formation of a thin layer of morphologically wild‐type cells covering the entire surface of the glass vials that is in contact with the culture medium (Figure 2A). This again introduces a strong bias, as these cells are not diluted to the same extent than the rest of the population. Stronger mixing of the cultures through increased air flow was not sufficient to prevent the formation of this thin film (data not shown). Finally, while still significantly delayed compared with similar experiments in rich medium (Figure 1, Table 1), the appearance of branched and multiseptated cells was still observed. The proportion of these aberrant cells in the cultures increased at non‐reproducible rates in parallel experiments (Figure 2B).

We concluded that, while the addition of galactose appeared to have a positive effect on limiting the flocculation phenotype, it was not sufficient for sustaining long‐term experiments, as glass adherence, ring formation and accumulation of abnormal cells were still observed.

### Deletion of the flocculin‐encoding gene *gsf2* prevents glass adherence

In order to prevent the accumulation of cells on the walls of the culture vessels, we first treated the glass surface with trichloromethyl‐silane, which is commonly used as an anti‐sticking agent (Friend and Yeo, 2010; Borysiak *et al*., 2013). However, this did not have any effect on the formation of the cell ring (data not shown) and showed only a limited improvement of the glass adherence problem, delaying the appearance of the film of cells by about 3 days. We then considered the use of various compounds that can be directly added to the culture medium and that are known to limit cell–cell and cell–surface adherence, including phthalate (Guthrie, 2002), EDTA (Matsuzawa *et al*., 2013) and a biocompatible non‐ionic detergent often used in mammalian cell culture, Pluronic. However, when assessing the effects of these chemicals on cells grown in standard batch cultures in EMM6S + 3% galactose at 32°C, we observed a range of phenotypes that precluded their use in the ministat experiments (Table 2). Addition of phthalate or EDTA resulted in significant changes in both generation time and length at division. While the use of Pluronic appeared to have limited effects on the cells, it proved incompatible with the way the populations in the ministats are mixed (air bubbling), as it results in rapid formation of foam in the tubes (data not shown).

**Table 2:** Growth of fission yeast cells in test media

**Medium**	**Generation time** **(min)**	**Size at division** **(μm)**
EMM6S	146.5 ± 1.0	13.8 ± 0.2
Pluronic 0.05%	138.7 ± 1.6	13.3 ± 0.1
Pluronic 0.1%	140.6 ± 2.5	12.8 ± 0.5
EDTA 0.1 mM	228.7 ± 5.1	9.9 ± 0.4
EDTA 1 mM	239.0 ± 0.0	10.8 ± 0.3
Phthalate 20 mM	168.9 ± 1.6	13.3 ± 0.2
Phthalate 40 mM	187.6 ± 0.9	15.8 ± 0.2

All media are based on EMM6S to which specific compounds were added as indicated. Experiments were carried out at 32°C in batch cultures. Standard errors of three independent experiments are shown. For cell size at division, *n* > 50 for each independent experiment.

We next took a genetic approach to the problems of adherence and ring formation and deleted the flocculin‐encoding gene *gsf2*. The flocculin Gsf2 mediates cell–cell adhesion through recognition of surface galactose residues, playing a role in non‐sexual flocculation as well as in adhesion and filamentous growth (Matsuzawa *et al*., 2011). In standard batch cultures (32°C in EMM6S or EMM6S + 3% galactose), *gsf*2Δ cells showed no phenotype (Figure 2C). Importantly, when these cells were grown in the ministat setups in EMM6S at 32°C, we observed neither ring formation nor glass adherence (Figure 2D). This indicated that deletion of *gsf2* could allow for long‐term assays with *S. pombe* cells in the ministat setups. In these conditions, however, we still observed a low percentage of multiseptated and branched cells after 4–5 days, which took over the entire culture after 22 days (Figure 2D). Experiments using *gsf*2Δ cells in the presence of 3% galactose showed similar results (Figure 2D).

Taken together, these data suggested that combining a deletion of the *gsf2* gene with the addition of galactose to the medium may be adapted to perform assays that require the maintenance of fission yeast cultures in exponential growth for many generations, without flocculation and glass adherence. However, this approach still presented strong limitations, as none of these improvements were able to prevent the appearance and accumulation of aberrant cells in the ministat cultures.

### Enhanced mixing limits the accumulation of aberrant cells

To address the presence of aberrant cells in the cultures, we considered the basic design of the ministats. We reasoned that the appearance and accumulation of multiseptated, branched cells over time may be favoured by the way the ministat culture setups were built. Indeed, cells that are discarded through the effluent line come from the surface of the liquid, which is in contact with the effluent needle. Insufficient mixing in the ministat vessels may introduce a non‐homogenous, size‐based distribution of the cells along the liquid column, potentially resulting in the preferential removal of smaller‐sized cells. To test this, we maintained *gsf*2Δ cells growing in standard glass flasks under strong shaking (EMM6S 3% galactose at 32°C), manually diluting the cultures at regular time intervals to prevent cell cycle exit. After 9 days, we did not detect any aberrant cells. This prompted us to change the type of vessels used for the ministats, switching to wider vials. This allowed us to grow similar volumes of cultures but with lower liquid columns (Figure 3A), potentially limiting size‐selectivity. In addition, we moved from the air‐mediated mixing of the population to the use of individual stir bars in each vial, and the temperature of the samples was maintained by immersing the entire system in a temperature‐controlled water bath (Figure 3B, C). Using this new version of the ministat devices, we ran long‐term cultures of *gsf*2Δ in EMM6S 3% galactose at 32°C. In these conditions, we did not observe flocculation or adherence to the vessel walls, but low percentages of aberrant cells started to appear after 13 days. Thus, while this represented a clear improvement over the initial setup, it remained insufficient for performing longer assays.

Interestingly, our initial experiments comparing the behaviours of these cultures in YE4S and EMM6S (Figure 1) showed that multiseptated branched cells appeared much earlier in YE4S than in EMM6S. We therefore hypothesized that the growth rate of cells may have an influence on this phenotype, with faster growing cells being more prone to displaying these defects in continuous culture systems (in batch cultures, the generation times of wild‐type cells in YE4S and EMM6S at 32°C are 121.6 ± 1.2 and 146.5 ± 1 min, respectively; the errors indicated are standard errors of three independent experiments). To test this, we repeated these experiments at 28 instead of 32°C, thus increasing the division time. Moreover, we reduced the length of the outer part of the effluent needle in order to avoid accumulation of cells in this part of the setup. In these conditions, we observed no phenotype and could not detect any aberrant cells after 23 days of continuous culture (Figure 3D). While this identified a set of modifications that appeared to be essential for long‐term cultures of fission yeast cells in these setups, all of the improvements were made sequentially. We therefore tested whether some of the alterations that we implemented were in fact dispensable in our latest system. Wild‐type cells were grown in the culture device at 28°C under strong mixing in EMM6S with or without galactose. Interestingly, we observed a significant delay in the appearance of the flocculation and ring phenotypes in EMM6S as well as a reduced, although still visible, adhesion to the glass walls in the presence of galactose (Table 3). When testing *gsf2Δ* cells in EMM6S, flocculation was apparent after 14 days, in contrast to the results presented in Figure 2D (Table 3). This suggests that flocculation in the absence of Gsf2 may be variable. Importantly, we never observed this phenotype in our long‐term laboratory evolution experiments when combining *gsf2Δ* with galactose (data not shown), demonstrating that both modifications are required for robust assays.

**Table 3:** Minimal set of conditions for growth of fission yeast cells in the final setup

**Strain**	**Medium**	**Flocculation**	**Day**	**Ring**	**Day**	**Wall adhesion**
WT	EMM6S	+	12	+	14	*n.d.*
WT	EMM6S + Gal	‐	*n.a*	‐	*n.a.*	+
*gsf2Δ*	EMM6S	+	14	‐	*n.a.*	‐

Experiments were performed in the setups in Figure 3(B) at 28°C in EMM6S or EMM6S +3% galactose as indicated. For the flocculation phenotype, the indicated days correspond to the first detection of flocs by microscopy. n.a., Not applicable; n.d., not determined. The wall adhesion phenotype was scored at the end of the experiments. All experiments were carried out for 16 days. A few multiseptated cells were observed in the WT strain grown in the presence of galactose at day 14.

Taken together, these data show that the use of ministat arrays for long‐term cultures can be adapted to *S. pombe*. For relatively short experiments (~10 days), deletion of the flocculin and addition of galactose are not essential when using our final system, showing its versatility and ease of use. However, long‐term experiments require addition of galactose to the medium, deletion of the flocculin‐encoding gene *gsf2*, efficient mixing of the population and slight reduction of the overall growth rate through the use of lower temperatures. Using this system, we have routinely performed experimental evolution assays using mutant cells of various initial generation times over more than 100 days (>500 generations, our unpublished data).

### Independent pump systems for the control of individual cultures

One remaining drawback of these arrays of ministats is that the dilution of each individual culture relies on a single, multichannel peristaltic pump. As a consequence, parallel assays using strains with very different generation times or using different conditions that affect the growth rate differentially are difficult to control. Previously, the standard and most efficient way of solving this issue in the ministat setups was to adapt the inner diameter of the dilution tubing for each vial to the generation time of the corresponding cultures, as well as to adjust the total volume of each culture to modulate the effective dilution rates individually. While this allows for the equilibration of the different cultures with one another, it remains time‐consuming, laborious and insufficiently robust. We therefore set out to design a cost‐effective, reliable and easy‐to‐build system in which each vessel depends on a single, independent pump (Figure 4A). To this end, we took advantage of small and autoclavable piezo pumps. Compared with standard small single‐channel peristaltic pumps and syringe pumps (Takahashi *et al*., 2015), these systems have the advantage of offering a broader range of flow rates and a more accurate control over the pumping rates. They are also easy to use and of similar costs.

The use of these systems required the design and fabrication of an electronic controller integrating the manufacturer‐provided modules for driving the pumps (Figure 4B, C; see Materials and Methods section). This allowed us to modulate both the amplitude and frequency of the pump operation for fine‐tuning the dilution rates. Importantly, by adding fluidic resistances of various inner diameters and lengths downstream of the pumps, we could cover a wide range of flow rates that were perfectly adapted to what is needed for the dilution of fission yeast cells (from 20 to 100 μl/min for cultures of 20 mL; Figure 4D). Interestingly, much higher flow rates can also be achieved (Figure 4D) and such pumps can be assembled in parallel, suggesting that our system can be used for other applications requiring faster dilution of the cells or higher culture volumes.

**Figure 5:**
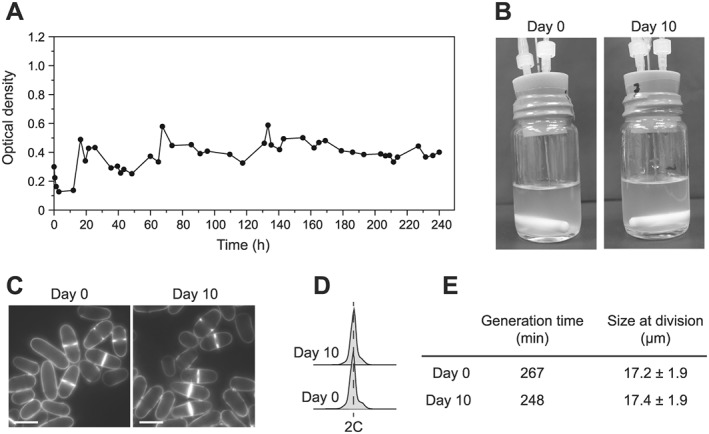
Growing *Schizosaccharomyces japonicus* in the culture system. S. japonicus cells were grown in the final setup in EMM6S + 3% galactose at 28°C. (A) Optical density measurements over a 10 day experiment, showing stabilization of the culture. (B, C) Pictures of the vials and cells at days 0 and 10. We observed no apparent flocculation or abnormal phenotypes. Scale bar = 10 μm. (D) Flow cytometry of cells as in (B) and (C). (E) Generation time and size at division of cells as in (B) and (C). For the generation time, a representative measurement is shown (second independent measurement: 277 and 267 min at days 0 and 10, respectively). For size at division, the standard deviation is shown (*n* = 100).

As a proof of concept, we grew in parallel two strains with very different generation times: one operating with a minimal CDK system consisting of a fusion between cyclin B Cdc13 and Cdk1/Cdc2 in the absence of the endogenous cell cycle control network (Coudreuse and Nurse, 2010; referred to as *MCN gsf2*Δ – minimal cell cycle network; batch culture generation time in EMM6S + 3% galactose at 28°C, 194 ± 2 min; standard error of three independent experiments is shown); and one operating with a similar circuit but lacking the well‐described Wee1/Cdc25 feedback loop on Cdc2 activity through mutation of the Wee1 target residues (Coudreuse and Nurse, 2010; T14A Y15F, referred to as *MCNAF gsf2*Δ; batch culture generation time in EMM6S + 3% galactose at 28°C, 281 ± 4 min; standard error of three independent experiments is shown). Importantly, in contrast to the initial ministat setup based on a multichannel peristaltic pump, we used identical culture volumes for both strains and only modulated the dilution rate through individually controlling the pump parameters. Strikingly, after the initial 48 h usually needed in all ministat experiments to stabilize the cultures and dilution rates, we could maintain both strains at constant density using different pump parameters (Figure 4E), compensating for any changes by modulating the flow rates. This was achieved without changing the culture volumes, positions of the effluent needles or characteristics of the dilution tubes. These results validate our system for the use of normalized growing conditions in long‐term cultures, limiting biases when comparing strains or conditions. In addition, we further validated this setup for growing the larger relative yeast species *Schizosaccharomyces japonicus*, using a wild‐type strain in the most optimal conditions identified for *S. pombe* (EMM6S + galactose, 28°C; Figure 5).

## Conclusion

We have adapted the original ministat setup initially optimized for S. cerevisiae for the use of *S. pombe*. The features of fission yeast cells, in terms of physiology, shape and size, made the standard ministats incompatible with long‐term experiments when exponential growth must be maintained. Altering both the system (type of medium, vial shape, mixing procedure, temperature) and the genetic background of the cells, we demonstrate that these setups represent a powerful approach for multiplexed long‐term experiments using fission yeast, including competition and experimental evolution assays. In addition, we have integrated a novel piezo pump‐based system to the ministats for affordable, easy and robust control of the dilution rates of individual vessels, thereby providing greater versatility and precision to the setup. The very broad range of flow rates offered by these pumps also suggests that our device may be used for a number of alternative applications, even those requiring large volumes and high dilution rates. While we have used and validated this setup as a basic manual turbidostat, adapting the dilution rates of the cultures to maintain their densities, feedback from optical density measurements would allow for a fully automated system. Finally, in its current configuration, this device could be used as a chemostat array through adjustment of the growth media.
